# Preparation of Structure Vacancy Defect Modified Diatomic‐Layered g‐C_3_N_4_ Nanosheet with Enhanced Photocatalytic Performance

**DOI:** 10.1002/advs.202302503

**Published:** 2023-06-21

**Authors:** Tian Liu, Wei Zhu, Ning Wang, Keyu Zhang, Xue Wen, Yan Xing, Yunfeng Li

**Affiliations:** ^1^ College of Environmental and Chemical Engineering Xi'an Key Laboratory of Textile Chemical Engineering Auxiliaries Xi'an Polytechnic University Xi'an 710048 P. R. China; ^2^ School of Chemistry Xi'an Jiaotong University Xi'an 710049 P. R. China; ^3^ Jilin Provincial Key Laboratory of Advanced Energy Materials Department of Chemistry Northeast Normal University Changchun 130024 P. R. China

**Keywords:** exciton density, g‐C_3_N_4_, photocatalysis, ultrathin structure, vacancy defect

## Abstract

Structure self‐modification of graphitic carbon nitride (g‐C_3_N_4_) without the assistance of other species has attracted considerable attention. In this study, the structure vacancy defect modified diatomic‐layered g‐C_3_N_4_ nanosheet (VCN) is synthesized by thermal treatment of bulk g‐C_3_N_4_ in a quartz tube with vacuum atmosphere that will generate a pressure‐thermal dual driving force to boost the exfoliation and formation of structure vacancy for g‐C_3_N_4_. The as‐prepared VCN possesses a large specific surface area with a rich pore structure to provide more active centers for catalytic reactions. Furthermore, the as‐formed special defect level in VCN sample can generate a higher exciton density at photoexcitation stage. Meanwhile, the photogenerated charges will rapidly transfer to VCN surface due to the greatly shortened transfer path resulting from the ultrathin structure (≈1.5 nm), which corresponds to two graphite carbon nitride atomic layers. In addition, the defect level alleviates the drawback of enlarged bandgap caused by the quantum size effect of nano‐scaled g‐C_3_N_4_, resulting in a well visible‐light utilization. As a result, the VCN sample exhibits an excellent photocatalytic performance both in hydrogen production and photodegradation of typical antibiotics.

## Graphical abstract

The vacancy defect modified diatomic‐layered g‐C_3_N_4_ nanosheets are prepared by thermal treatment of bulk g‐C_3_N_4_ in a quartz tube with vacuum atmosphere. The prepared photocatalyst exhibits an excellent photocatalytic performance both in hydrogen production and photodegradation of typical antibiotics due to the special role of defect and ultrathin structure in increasing exciton density and carrier transfer.

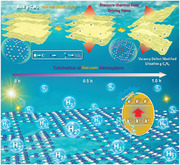

## 1 Introduction

Photocatalytic technology has aroused great research interest as it provides a promising solution to serious environmental and energy issues.^[^ ^1^, ^2^, ^3^, ^4^, ^5^ ^]^ Photocatalysts can transform solar energy into chemical energy that is used in various fields, such as pollutant degradation,^[^ ^6^, ^7^, ^8^ ^]^ hydrogen production,^[^ ^9^, ^10^, ^11^ ^]^ CO_2_ reduction^[^ ^12^, ^13^, ^14^ ^]^ and nitrogen fixation.^[^ ^15^, ^16^, ^17^ ^]^ Up to now, a large number of materials have been developed and applied to achieve improvement in photocatalytic performance.^[^ ^18^, ^19^, ^20^, ^21^, ^22^, ^23^, ^24^ ^]^ However, the rational design of sustainable and efficient visible‐light photocatalyst is still the most challenging task.^[^ ^25^, ^26^, ^27^, ^28^, ^29^ ^]^ For example, only when the conversion efficiency from solar to hydrogen (STH) of photocatalysts reaches over 10%, can they be competitive compared to traditional hydrogen production technology. Currently, the low‐cost metal‐free photocatalyst with tunable band structure is widely developed in photocatalytic energy conversion due to its sustained availability and economic adaptability.^[^ ^30^, ^31^, ^32^, ^33^, ^34^ ^]^ Among them, graphitic carbon nitride (g‐C_3_N_4_) has received great attention due to the groundbreaking work by Wang et al.^[^ ^35^ ^]^ As a *π*‐conjugated metal‐free polymer, g‐C_3_N_4_ exhibits a suitable band structure, simple preparation, and excellent structure stability. However, the inherent drawbacks of g‐C_3_N_4_, including small specific surface area, inefficient visible‐light absorption, and rapid charges recombination, greatly limit its photocatalytic performance.

In order to overcome the above drawbacks, researchers have developed many strategies to improve the photocatalytic performance of g‐C_3_N_4_, such as elemental doping,^[^ ^36^, ^37^, ^38^ ^]^ morphology modulation,^[^ ^39^, ^40^, ^41^ ^]^ constructing heterojunction,^[^ ^42^, ^43^, ^44^ ^]^ developing defect engineering,^[^ ^45^, ^46^, ^47^ ^]^ etc. Among these methods, defect functionalization on g‐C_3_N_4_ structure is a common and feasible strategy for improving photocatalytic conversion efficiency. Introducing defects into 2D g‐C_3_N_4_ framework significantly enhances its photocatalytic activity through optimizing the electronic band structure to improve the optical utilization ability and carrier transfer kinetic. Yu et al.^[^ ^48^ ^]^ fabricated g‐C_3_N_4_ with nitrogen vacancy by one‐pot thermal polymerization of urea and fumaric acid. The result shows that g‐C_3_N_4_ with nitrogen vacancy could generate more photoexcited electrons and raise the H_2_O molecule adsorption capacity. Zhao et al.^[^ ^49^ ^]^ calcined the mixture of g‐C_3_N_4_ and sodium borohydride in an inert atmosphere, and then boron doping and nitrogen vacancy were simultaneously achieved into g‐C_3_N_4_. The as‐obtained boron‐doped and nitrogen‐vacancy g‐C_3_N_4_ displays an efficient photocatalytic performance for oxygen evolution with a rate of 561.2 µmol h^−1^ g^−1^. Zhang et al.^[^ ^50^ ^]^ successfully prepared the sulfur‐doped and nitrogen‐vacant g‐C_3_N_4_ by annealing g‐C_3_N_4_ in S steam. The photocatalytic H_2_ production rate of prepared sample under visible‐light irradiation reaches 5192.2 µmol h^−1^ g^−1^. It can be seen that defect engineering is widely used to optimize the band structure of g‐C_3_N_4_ to improve the photocatalytic performance. Up to now, most of work focuses on utilization of templates or special substances to construct the structure defects in g‐C_3_N_4_, while it is still a challenge to introduce vacancy defects only by regulating the molecular structure of g‐C_3_N_4_ without the assistance of other species.

In this paper, the structure vacancy defect modified ultrathin g‐C_3_N_4_ nanosheet (VCN) is synthesized by thermal treatment of bulk g‐C_3_N_4_ (GCN) in a quartz tube with vacuum atmosphere. This special condition will generate a pressure‐thermal dual driving force to boost the exfoliation of g‐C_3_N_4_ and formation of structure vacancy. The as‐prepared VCN has a larger specific surface area with an improved pore structure that provides adequate active sites for surface reaction. Notably, the as‐formed special defect level in VCN sample can generate a higher exciton density at the photoexcitation stage. Meanwhile, the photogenerated charges will rapidly transfer to VCN surface due to the greatly shortened transfer path resulting from the reduced catalyst size. In addition, the defect level alleviates the drawback of enlarged bandgap caused by the quantum size effect of nano‐scaled g‐C_3_N_4_, resulting in a well‐visible‐light utilization. As a result, the VCN sample shows an excellent photocatalytic performance in hydrogen production and photodegradation of typical antibiotics, ciprofloxacin (CIP), and tetracycline hydrochloride (TC).

## 2 Results and Discussion

### 2.1 Structure and Property Analysis

**Figure 1:**
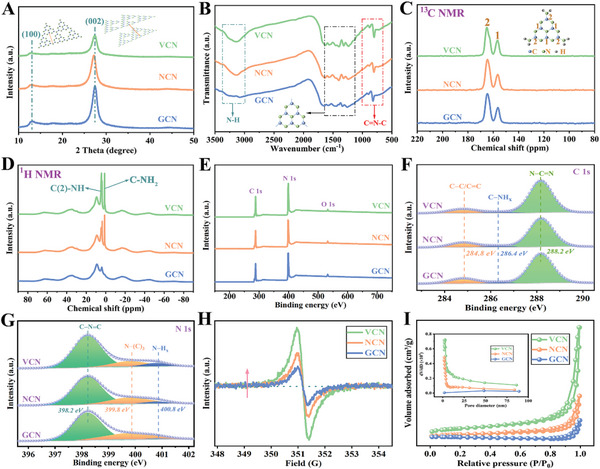
A) XRD patterns, B) FT‐IR spectra, (C) solid‐state ^13^C NMR spectra, and D) solid‐state ^1^H NMR spectra of as‐prepared samples; XPS survey spectra E) high‐resolution spectra of C 1 s F) and N 1 s G) of as‐prepared samples; H) EPR spectra and I) N_2_ adsorption–desorption isotherms and pore size distribution curves of as‐prepared samples.

As presented in **Figure** **1**A, X‐ray diffraction (XRD) patterns of all photocatalysts reveal the formation of graphite‐like stacking C_3_N_4_ layers. The diffraction peaks for as‐prepared samples appear at 13.1° and 27.4°, and the low angle diffraction peak is attributed to the (1 0 0) plane, corresponding to tri‐s‐triazine units of g‐C_3_N_4_, while the high angle diffraction peak comes from the (0 0 2) plane, which is ascribed to the interlayer *π‐π* stacking.^[^ ^51^, ^52^, ^53^ ^]^ The XRD peaks for VCN become weaker compared to the GCN and NCN samples, which is mainly due to more structure defects and decreased thickness of VCN caused by pressure‐thermal dual driving forces during the preparation process.^[^ ^45^, ^54^, ^55^ ^]^ The synthesized GCN, NCN, and VCN were also characterized by Fourier transform infrared (FT‐IR) spectroscopy, as shown in Figure 1B. Specifically, the sharp breathing peak at 810 cm^−1^ of all the samples could be attributed to the triazine units, and the characteristic peaks between 1700 and 1200 cm^−1^ are associated with CN stretching vibration modes. The broad band between 3500 and 3000 cm^−1^ is the characteristic feature of N—H stretching.^[^ ^56^, ^57^ ^]^ Compared with GCN sample, a stronger N—H stretching vibration peak for VCN and NCN indicates more structure defects in these two samples. Figure 1C shows the solid‐state ^13^C CP‐MAS‐NMR spectra of GCN, NCN, and VCN samples. It can be seen that all the photocatalysts appear to have two distinct peaks at 157.2 and 165.6 ppm belonging to the characteristic C(1) atoms of N=C—N_2_ and C(2) atoms of N=C—N (NH_x_) in the heptazine units, respectively. The ratio of C(2) to C(1) atoms increases from 2.21 and 2.35 in GCN and NCN samples, respectively to 2.47 in VCN sample, indicating more defect vacancies in VCN. In addition, all the samples were further studied by solid‐state ^1^H CP‐MAS‐NMR, as shown in Figure 1D. It can be seen that the peak at 9.05 ppm in all the samples is ascribed to strong hydrogen bonds between tri‐s‐triazine units, or interlayer stacking. The two peaks at 1.03 and 3.61 ppm present in all samples assign to C—NH_2_ and C(2)—NH, respectively.^[^ ^58^ ^]^ The intensity of these two peaks in VCN greatly increases, which further reveals the generation of defect vacancies during the preparation process. The XPS spectra of GCN, NCN, and VCN samples were also studied to obtain the surface chemical compositions. The XPS survey spectrum in Figure 1E indicates that all the samples mainly consist of C and N elements. The C 1s XPS spectrum of photocatalysts contains three peaks located at 288.2, 286.4, and 284.8 eV, corresponding to the N—C=N in aromatic rings, C—NH_x_ groups, and adventitious carbon, respectively (Figure 1F).^[^ ^59^ ^]^ The content of C—C/C=C in the sample decreases in descending order from GCN to VCN, which originates from decomposition of small organic molecules. On the contrary, the sequential increase of C—NH_x_ peak intensity from GCN to VCN is attributed to the break of N—(C)_3_ bond and the exfoliation of g‐C_3_N_4_ layers to generate structure defect and ultrathin structure (Figure S1, Supporting Information). The N 1s XPS spectrum of all the samples is composed of three binding peaks at 398.2, 399.8, and 400.8 eV, which is attributed to bi‐coordinated N, tri‐coordinated N and NH_x_ groups in the melon framework, respectively (Figure 1G).^[^ ^60^ ^]^ Apparently, the characteristic peak of N—(C)_3_ decreases from GCN to VCN in order, while the characteristic peak of NH_x_ increases from GCN to VCN, which reveals a similar result with C 1s XPS. Furthermore, the electron paramagnetic resonance (EPR) of all the samples (Figure 1H) indicates that the VCN sample shows a much stronger EPR signal than that of GCN and NCN, signifying a large number of surface structure defects. The results of XRD, FT‐IR spectra, XPS spectra, solid‐state ^13^C CP‐MAS‐NMR, solid‐state ^1^H CP‐MAS‐NMR, and EPR spectroscopy have sufficiently demonstrated that the as‐prepared VCN sample possesses the vacancy defect in its molecular structure.

In order to study the pore structure and adsorption ability of as‐prepared photocatalysts, we employ the N_2_ adsorption–desorption tests to investigate Brunauer–Emmett–Teller (BET) specific surface area and pore size distribution of GCN, NCN, and VCN samples (Figure 1I). It is noted that all the samples possess a similar shape of N_2_ adsorption–desorption isotherm that could be assigned to type IV isotherms with H_3_ hysteresis loops. Obviously, the isotherm of VCN has a larger amount of nitrogen adsorption at high relative pressure than that of GCN and NCN, illustrating that the vacancy defect and ultrathin structure of VCN can greatly enhance the BET specific surface area. Thus, the VCN sample shows a large BET specific surface area of 59.8 m^2^ g^−1^, which is 2.8 and 10.9 times higher than NCN and GCN samples, respectively (Table S1, Supporting Information). Furthermore, the pore size distribution using Barrett‐Joyner‐Halenda (BJH) model (inset of Figure 1I) shows that VCN possesses a rich pore structure that results from the structure defect.

**Figure 2:**
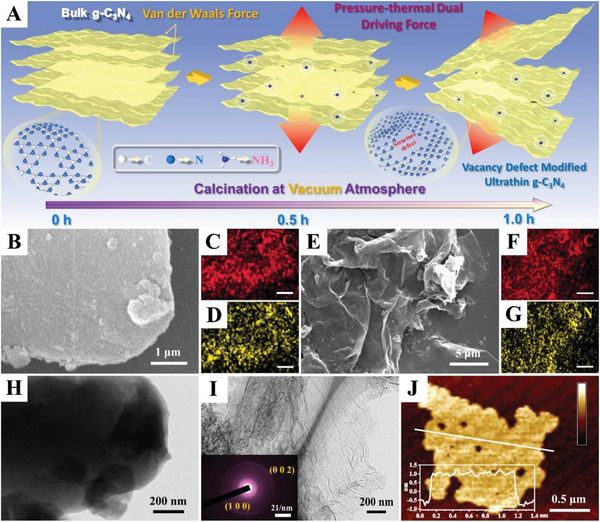
A) Schematic illustration for the formation of VCN sample; B) SEM image, and c, D) element mappings of GCN; E) SEM image and F, G) element mappings of VCN; TEM images of H) GCN and I) VCN; J) AFM image of VCN sample.

The schematic illustration for the formation of VCN sample is shown in **Figure** **2**A. The vacancy defect and ultrathin structure can be introduced synchronously by thermal treatment of bulk g‐C_3_N_4_ in a quartz tube with vacuum atmosphere. This special condition will generate a pressure‐thermal dual driving force to boost the exfoliation of g‐C_3_N_4_ and formation of structure vacancy. For the as‐prepared GCN, NCN, and VCN samples, their microscopic morphology has been studied by scanning electron microscopy (SEM) and transmission electron microscopy (TEM) images. As shown in Figure 2B, the SEM image of GCN sample shows a typical thick 2D sheet structure consisting of two primary elements of C and N (Figure 2C,D), which is consistent with its TEM image (Figure 2H). The SEM and TEM images in Figure S2 (Supporting Information) indicate that the thickness of NCN sample has not decreased significantly compared with GCN. Nevertheless, the VCN sample shows an ultrathin nanosheet structure similar to graphene (Figure 2E,I), and its element composition has not changed (Figure 2F,G). The inset of Figure 2I shows that VCN exhibits the typical diffraction rings corresponding to (1 0 0) and (0 0 2) planes. In addition, the atomic force microscope (AFM) image in Figure 2J indicates that thickness of VCN sample is about 1.5 nm, which corresponds to the two graphite carbon nitride atomic layers. The TEM and SEM results indicate that the pressure‐thermal dual driving force generated in synthesis process could boost the exfoliation of g‐C_3_N_4_ through overcoming the interlayer van der Waals force.

**Figure 3:**
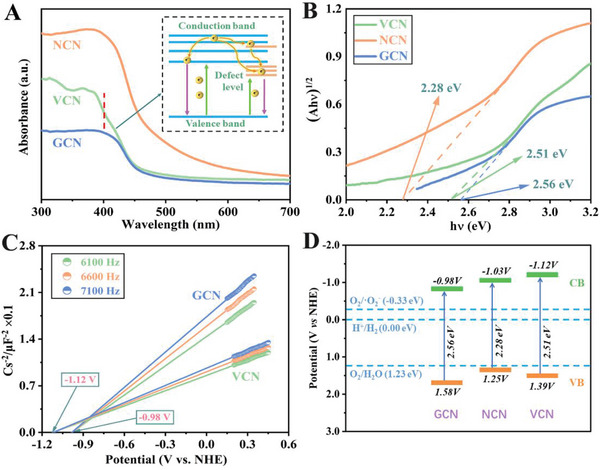
A) UV–vis DRS and B) Kubelka–Munk plots of as‐prepared samples; C) Mott–Schottky plots of GCN and VCN samples; D) Band structure of as‐prepared GCN, NCN, and VCN samples.

The optical properties of all the as‐prepared samples were investigated by UV–vis diffuse reflectance spectra (UV–vis DRS) (**Figure** **3**A). It can be seen that all the samples show an obvious visible light absorption. The light absorption range of NCN sample greatly redshifts compared to GCN due to structure defect generated during thermal treatment in N_2_. Notably, the VCN sample shows a significantly reduced thickness observed in TEM and SEM, which usually causes a broadening bandgap due to the well‐known quantum size effect. However, the optical property of VCN does not change obviously compared to GCN, which is attributed to the significant role of structure defect in semiconductor photoexcitation process (inset in Figure 3A). The bandgap (Eg) of all photocatalysts has been calculated in Figure 3B. According to the Kubelka–Munk function, the Eg of GCN, NCN, and VCN is about 2.56, 2.28, and 2.51 eV, respectively. Meanwhile, the flat‐band potentials of all the samples are obtained via extrapolating to the x‐intercept in Mott–Schottky plot. As shown in Figure 3C and Figure S3 (Supporting Information), the positive slope of Mott–Schottky plot suggests that all the samples are typical n‐type semiconductor characteristics.^[^ ^61^ ^]^ The conduction band (CB) potentials have been tested to be about −0.98, −1.03, and −1.12 V (vs NHE) for GCN, NCN, and VCN, respectively. Based on the above characterization results and the formula (*E*_VB_  = *E*_g_ + *E*_CB_),^[^ ^62^, ^63^ ^]^ the valence band (VB) potentials of GCN, NCN, and VCN are calculated to be about 1.58, 1.25, and 1.39 V (vs NHE), respectively. The obtained band structures of GCN, NCN, and VCN has been shown in Figure 3D.

**Figure 4:**
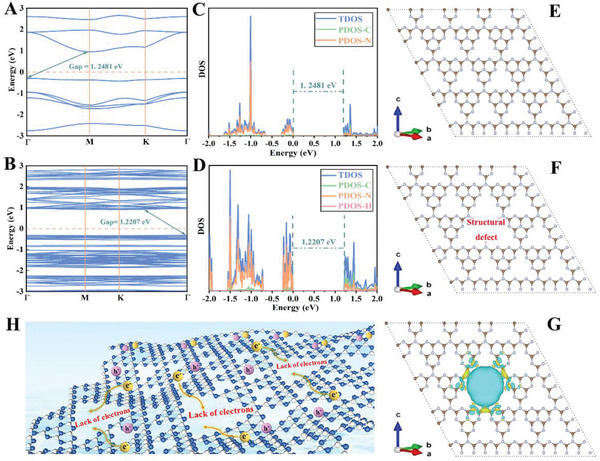
Calculated band structure of A) GCN and B) VCN samples; Density of states of C) GCN and D) VCN samples; Established structure models of E) GCN and F) VCN samples; G) Calculated differential charge distribution of VCN (yellow and blue colors indicate the increase and decrease of electron distributions, respectively); H) Schematic diagram of charge transfer for VCN.

To further understand the effect of structural vacancy defect on band structure and charge transfer, the density functional theory (DFT) of as‐prepared samples has been implemented. As shown in **Figure** **4**A,B, the results of DFT indicate that both VCN and GCN show an indirect semiconductor characteristic, and introducing structure defects can indeed narrow the bandgap of g‐C_3_N_4_. The bandgap of prepared VCN decreases to 1.2207 eV compared to 1.2481 eV of GCN. The density of states (DOS) of GCN and VCN have also been calculated. As exhibited in Figure 4C, both nonlocal C p_x_ and N p_x_ orbits form the VB top of GCN, while the CB bottom is consisted of hybrid C p_x_ and N p_y_ orbits. The VCN sample modified by vacancy defect displays similar results (Figure 4D). To further reveal the essential relationship between the structure vacancy defect and photogenerated carriers transfer, we employ the charge density distributions of VCN sample. The established structure models of GCN and VCN are displayed in Figure 4E,F. It can be seen that the presence of defective structure results in a more localized spatial distribution of charge density, and vacancy position in VCN sample shows a significantly decreased electron distribution (Figure 4G). Under visible light irradiation, this special localized charge redistribution will generate an internal electric field, thus increasing the separation efficiency of photoexcited electrons and holes. The specific charge transfer path in prepared VCN sample is proposed, and as shown in Figure 4H, the photogenerated electrons tend to transfer to electron depletion region and the photogenerated holes tend to transfer to electron accumulation region, respectively, to increase the separation efficiency.

### 2.2 Photocatalytic Activity

**Figure 5:**
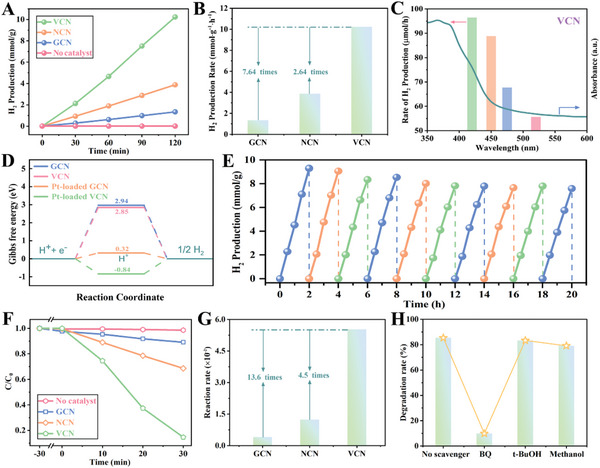
A) Hydrogen evolution performance of as‐prepared samples; B) Comparison of hydrogen production rate for as‐prepared samples; C) Hydrogen production at different wavelengths for VCN; D) Calculated Gibbs free‐energy diagram of H_2_ production reaction; E) Cycling test of photocatalytic H_2_ production for VCN. F) Photocatalytic degradation of CIP for GCN, NCN, and VCN photocatalysts; G) Comparison of degradation rate of CIP for as‐prepared samples; H) Trapping experiment of CIP by using VCN sample.

The photocatalytic activities of the GCN, NCN, and VCN photocatalysts were assessed by hydrogen production and degrading antibiotics of CIP and TC. Photocatalytic hydrogen production over as‐prepared photocatalysts loaded with 1 wt.% Pt as cocatalyst is evaluated under a 300 W Xe lamp (*λ* > 420 nm, Merry Change, MCPF300B) by using the TEOA as a hole scavenger. As illustrated in **Figure** **5**A, the amount of H_2_ evolution for GCN, NCN, and VCN is about 1.34, 3.88, and 10.24 mmol g^−1^ under light irradiation for two hours, respectively. The VCN sample shows the highest H_2_ evolution rate of 5.12 mmol h^−1^ g^−1^, which is 7.64 and 2.64 times higher than that of GCN and NCN samples, respectively (Figure 5B). The hydrogen production of VCN is also used to study the effect of light irradiation under different wavelengths (420, 450, 475, and 520 nm) (Figure 5C), and it can be seen that the amount of H_2_ conforms to the light absorption characteristics of VCN sample. The apparent quantum efficiency (AQE) of VCN has been tested to be 12.2% at 420 nm, which is much higher than that of GCN (2.51%) and NCN (4.24%) samples (Figure S4, Supporting Information). DFT calculations are also used to investigate the special role of vacancy defects in photocatalytic hydrogen evolution reactions (Figure 5D; Tables S2–S5, Supporting Information). Based on the optimized hydrogen adsorption model, the Gibbs free energy (ΔG) of GCN and VCN for total hydrogen evolution reaction is about 2.94 and 2.85 eV, respectively. After loading Pt cocatalyst, the ΔG of as‐prepared samples greatly reduces, and the Pt‐loading VCN sample shows the lowest ΔG value, signifying an optimized intermediate reaction process in thermodynamics. The photocatalytic H_2_ production rate of VCN does not display a significant decline after ten cycles illustrating its excellent structure stability (Figure 5E; S5, Supporting Information). As shown in the attached video, the intense hydrogen bubbles can be directly observed during the testing process.

In the degradation experiment, we should achieve the adsorption–desorption equilibrium between the as‐prepared samples and antibiotics by continuous stirring before light illumination. After visible light irradiation for 30 min, 85%, 31%, and 11% of CIP are degraded in the presence of VCN, NCN, and GCN, respectively (Figure 5F). The degradation rate constant for VCN (0.055 min^−1^) is almost 13.6 times than that of GCN (0.004 min^−1^) (Figure 5G). To justify the major active species in the photocatalytic reaction, the trapping experiments for degradation of CIP and TC were implemented by employing methanol, tert‐butanol (t‐BuOH), and 1,4‐benzoquinone (BQ) as hole, ·OH and ·O^2−^ scavengers, respectively.^[^ ^64^, ^65^ ^]^ The trapping experiments indicate that ·O^2−^ is the major active specie during the process of CIP degradation by using VCN photocatalyst (Figure 5H). In addition, the VCN photocatalyst also shows an excellent photocatalytic activity in degradation of another antibiotic TC with ·O^2−^ as major active specie (Figure S6, Supporting Information).

### 2.3 Photocatalytic Mechanism

**Figure 6:**
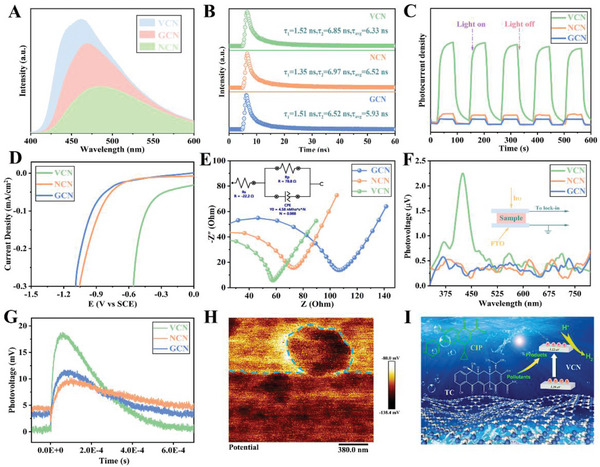
A) Steady‐state PL spectra, B) transient‐state PL spectra, C) photocurrent response, D) LSV curves, E) EIS spectra, F) SPV spectra, and G) TPV spectra of as‐prepared samples; H) KPFM image of VCN sample; I) Schematic diagram of photogenerated charge transfer and catalytic mechanism for VCN sample.

In order to further study the crucial role of ultrathin structure and structure vacancy in photogenerated charge transport, various photoelectric characterizations have been carried out. As observed in **Figure** **6**A, the NCN sample shows a weaker PL emission peak intensity than original GCN, indicating that the structure vacancy could effectively promote charge transfer by regulating the localized charge redistribution. Notably, the PL peak intensity has increased in VCN, sample mainly resulting from the increased exciton density in photoexcitation stage due to the synergistic effect of ultrathin structure and defect vacancy. This increased exciton density could be well demonstrated by steady‐state surface photovoltage and transient photovoltage (Figure 6F and G). The charge separation of prepared samples is also studied by time‐resolved PL spectra (TRPL). The variation trend of lifetime could be evidently observed from Figure 6B, and the average PL lifetime (*τ*) is determined by the following equation:

(1) $$\left(\tau \right)=\frac{A_{1}\tau_{1}^{2}+A_{2}\tau_{2}^{2}+A_{3}\tau_{3}^{2}\cdot \cdot \cdot }{A_{1}\tau_{1}+A_{2}\tau_{2}+A_{3}\tau_{3}\cdot \cdot \cdot }\;$$

The resulting PL lifetime (*τ*) of VCN and NCN is 6.33 and 6.52 ns, respectively, which is almost 1.07 and 1.09 times longer than that of GCN. Thus, the VCN sample has both high exciton density and long carrier lifetime, which greatly promotes the opportunity for photogenerated charges to participate in surface catalytic reactions. The transient photocurrent response has been developed to further evaluate the photogenerated carrier density and charge separation. As shown in Figure 6C, the photocurrent intensity of VCN sample is much larger than that of GCN and NCN samples, further indicating that the VCN sample possesses both high exciton density and fast charge separation. Furthermore, the linear sweep voltammetry (LSV) profiles of as‐prepared samples are exhibited in Figure 6D. It can be seen that the VCN sample possesses the lowest overpotential over the whole potential range, which is extremely beneficial to surface catalytic reactions. Meanwhile, the electrochemical impedance spectroscopy (EIS) of prepared samples is also measured. As observed in Figure 6E, VCN exhibits the smallest arc radius in semicircular Nyquist curves implying the most efficient charge transfer. The Nernst curve of VCN is well simulated with the electrical equivalent circuit model (inset in Figure 6E). The transmission characteristics of photogenerated carriers generated in prepared photocatalysts under illumination conditions are detected by surface photovoltage (SPV). As a typical n‐type semiconductor, the Fermi level of g‐C_3_N_4_ is higher than its surface state, thus the electrons will transfer from the bulk to surface until reaching equilibrium to form the internal electric field to promote the charge transfer. In Figure 6F, we can observe that the surface photovoltage responses of all samples are positive, indicating that all the prepared samples are n‐type semiconductors.^[^ ^54^, ^66^ ^]^ Notably, the VCN sample exhibits a much higher SPV signal than NCN and GCN samples, implying that more photogenerated excitons are generated in VCN sample and transfer to its surface, which could be also confirmed by transient photovoltage (TPV) characterization (Figure 6G). To further confirm the surface potential of VCN sample, we perform the spatially resolved potential analysis for VCN by using Kelvin Probe Force Microscopy (KPFM) (Figure 6H), and the result of KPFM is well consistent with SPV and TPV characterizations. Based on the above various photoelectric tests, we can see that the structure vacancy defect and ultrathin structure are beneficial for the photoinduced exciton formation and charge separation. The photocatalytic mechanisms for hydrogen evolution and photo‐degradation are illustrated in Figure 6I.

## 3 Conclusion

In summary, the structure vacancy defect modified ultrathin g‐C_3_N_4_ nanosheet with highly effective photocatalytic performance has been successfully fabricated. The as‐formed vacancy defect and ultrathin structure of VCN sample can generate a higher exciton density at photoexcitation stage, and then the photogenerated charges will rapidly transfer to VCN surface due to the greatly shortened transfer path and localized charge redistribution. Furthermore, the defect level also alleviates the drawback of enlarged bandgap caused by the quantum size effect of nano‐scaled g‐C_3_N_4_, resulting in a well‐visible‐light utilization. As a result, VCN shows a high photocatalytic hydrogen evolution rate of 5.12 mmol h^−1^ g^−1^, which is approximately 7.64 times higher than that of GCN. Meanwhile, the degradation of both TC and CIP by VCN could reach 85% in 30 min. This work combines the pressure‐thermal exfoliation and structure vacancy defects for modification of g‐C_3_N_4_ to improve its photocatalytic performance, thus providing a new insight for achieving sustainable energy in the future.

## 4 Experimental Section

### Preparation of Bulk g‐C_3_N_4_ (GCN)

Bulk g‐C_3_N_4_ was synthesized by direct condensation of melamine. In a typical synthesis, melamine was ground and placed into a covered alumina crucible, then thermally treated in air at 823 K for 2 h with a rate of 5 K min^−1^. After cooling down to room temperature, the yellow GCN was ground and collected for further use.

### Preparation of Structure Vacancy Defect Modified Ultrathin g‐C_3_N_4_ (VCN)

The structure vacancy modified ultrathin g‐C_3_N_4_ was prepared by thermal treatment of bulk g‐C_3_N_4_ in a quartz tube with vacuum atmosphere, and the typical process is as follows: 500 mg of GCN was placed into a porcelain boat, heated with 5 K min^−1^ to 863 K for 1 h in a quartz tube with vacuum degree at −0.098 MPa, and then cooled to room temperature. The sample obtained by thermal treatment GCN under N_2_ atmosphere (NCN) is used for comparison. Typically, 500 mg of as‐synthesized GCN was thermally treated at 863 K for 1 h at a heating rate of 5 K min^−1^ in nitrogen atmosphere, followed by cooling down to room temperature.

### Characterization

The crystalline structures of the photocatalysts were determined by X‐ray diffraction (XRD) using a Siemens D5005 Diffractometer with a Cu K*α* radiation source (*λ* = 1.5418  Å). Fourier transform infrared (FTIR) measurements from 4000 to 400 cm^−1^ were performed using a Spotlight 400 spectrometer. Solid‐state ^13^C and ^1^H magnetic resonance (NMR) spectra were recorded on a Bruker AVANCE III 400 MHz WB solid‐state NMR spectrometer at room temperature. X‐ray photoelectron spectroscopy (XPS) was performed using a Thermo Scientific K‐Alpha XPS instrument with a standard monochromatic light source. The room temperature electron paramagnetic resonance (EPR) spectra of the photocatalysts were measured by Bruker A300 spectrometer. N_2_ adsorption–desorption isotherms were carried out by a Micromeritics ASAP 2020 plus analyzer at 77.4 K. Scanning electron microscopy (SEM) images were performed on XL30 ESEM FEG microscope. Transmission electron microscopy (TEM) images were obtained using a JEM‐2100F microscope with an accelerating voltage of 200 kV. Mott–Schottky plot, electrochemical impedance spectroscopy (EIS), linear sweep voltammetry (LSV), and transient photocurrent response spectrum were conducted by using a standard three‐electrode cell with probe solution as the electrolyte, platinum as a counter, saturated calomel electrode as a reference electrode, and ITO glass deposited with sample as a working electrode. The surface photovoltage spectra (SPV) of samples were tested using a solid junction apparatus equipped with a light source‐monochromator‐lock‐in detection analyzer, and the samples were irradiated by a 500 W xenon lamp with a triple‐prism monochromator (Hiliger and Watt, D300). The photovoltage data was collected by an amplifier (Brookdeal, 9503‐SC). Transient photovoltage (TPV) was obtained by self‐assembled surface photovoltaic testing equipment. UV–vis diffuse reflectance spectra (UV–vis DRS) were tested using a Cary 500 spectrometer. Photo‐luminescence (PL) spectra of solid samples were measured using a FLSP920 Edinburgh Fluorescence Spectrometer. Time‐resolved photoluminescence (TRPL) measurements were carried out using time‐correlated single‐photon counting. The thickness and surface potential of VCN nanosheet were measured by Bruker Multimode 8 atomic force microscope (AFM).

### Computational Method

The PAW pseudopotentials implemented in the VASP were utilized for all DFT calculations. Exchange‐correlation functions were determined using the PBE generalized gradient approximation. This study treated the valence electron configurations of C(2s^2^2p^2^), N(2s^2^2p^3^), Pt(5d^9^6s^1^), and H(ultrasoft). The optimization structure was subject to energy and force convergence criteria per atom of 10^−5^ eV and 0.02 eV Å^−1^, respectively, with a cut‐off energy for plane wave basis set to 600 eV. To calculate the electronic structure more accurately, it employed the Monkhorst‐Pack scheme point mesh and used finer k‐point. For the modified g‐C_3_N_4_, it built a 4 × 4 large supercell of g‐C_3_N_4_ to ensure sufficient space between two neighboring groups due to the periodic structure. To eliminate periodic interaction between monolayer modified g‐C_3_N_4_, it set the vacuum thickness to 15 Å in the z‐direction. It is worth noting that in the modeling related to the cocatalyst Pt, it used zero valence rather than cation to represent the state of obtaining electrons from the excited state g‐C_3_N_4_ under illumination.

The formulas for calculating the Gibbs free energy change are as follows:

(2) $$\Delta G\left(H^{*}\right)\;=\;G\left(H^{*}/m-C_{3}N_{4}\right)-\frac{1}{2}G\left(H_{2}\right)-G\left(m-C_{3}N_{4}\right)$$

(3) $$G\left(298.15K\right)\;=\varepsilon_{ele}\;+ZPE+\Delta G_{0K\to 298.15K}$$

in which G(m‐C_3_N_4_), G(H_2_), G(H*/m‐C_3_N_4_) represent the Gibbs free energy of unmodified/modified g‐C_3_N_4_ with/without Pt as cocatalyst, gaseous hydrogen and H* absorbed unmodified/modified g‐C_3_N_4_ with/without Pt as cocatalyst, respectively. *ε*_ele_ denotes electronic energy calculated by DFT. ZPE symbolizes zero correction energy. Δ*G*_0*K* → 298.15*K*_represents Gibbs free energy correction from 0 to 298.15 K.

## Conflict of Interest

The authors declare no conflict of interest.

## Supporting information

Supporting Information

Supplemental Video 1

## Data Availability

The data that support the findings of this study are available from the corresponding author upon reasonable request.
