# Supplementary material for: Preparation of Structure Vacancy Defect Modified Diatomic‐Layered g‐C3N4 Nanosheet with Enhanced Photocatalytic Performance
Source: Adv Sci (Weinh). 2023 Jun 21;10(24):2302503. doi: 10.1002/advs.202302503 (PMC10460902; doi:10.1002/advs.202302503)
Supplement: Supplementary file 1 — Supporting Information [file ADVS-10-2302503-s001.pdf]

ADVANCED SCIENCE Open Access

Supporting Information

for *Adv. Sci.*, DOI 10.1002/advs.202302503

Preparation of Structure Vacancy Defect Modified Diatomic-Layered g-C_3N_4 Nanosheet with Enhanced Photocatalytic Performance

*Tian Liu, Wei Zhu, Ning Wang, Keyu Zhang, Xue Wen, Yan Xing and Yunfeng Li**

Supporting Information

Preparation of structure vacancy defect modified diatomic-layered g-C_3N_4 nanosheet with enhanced photocatalytic performance

Tian Liu,[†] *Wei Zhu,*[†] *Ning Wang, Keyu Zhang, Xue Wen, Yan Xing, Yunfeng Li**

Materials

Melamine ($C_3H_6N_6$, ≥99.0%) was purchased from Sinopharm Chemical Reagent Co., Ltd. Triethanolamine (TEOA, AR), chloroplatinic acid (H_2PtCl_6•$6H_2O$, 37.5%), ciprofloxacin ($C_{17}H_{18}FN_3O_3$, ≥98.0%), tetracycline hydrochloride ($C_{22}H_{25}ClN_2O_8$, 98%) and p-Benzoquinone ($C_6H_4O_2$, 99.0%) were all obtained from Aladdin (Shanghai, China). Tertiary butanol ($C_4H_{10}O$, AR) was purchased from Tianjin Comio Chemical Reagent Co., Ltd. Methanol (CH_3OH, 99.9%) was purchased from Tianjin Fuchen Chemical Reagent Factory. All chemical reagents were analytical grade and being used without further purification. Deionized water was utilized for all experiments.

Photocatalytic test

The photocatalytic H_2 evolution was carried out in a closed online system (Beijing Merry Change Technology Co.,Ltd) at ambient temperature. In a typical photocatalytic procedure, 30 mg of as-prepared sample was placed into a 50 mL aqueous solution using 5 mL of triethanolamine (TEOA) as sacrificial agent and H_2PtCl_6 as cocatalyst. The obtained catalyst suspension was ultrasonically treated and fully degassed before light irradiation. The amount of H_2 product was detection by a gas chromatography (TCD detector) with argon as carrier gas.

To evaluate the photocatalytic degradation efficiency of as-prepared samples, 30 mg of photocatalyst for degradation of typical antibiotic ciprofloxacin (CIP, 10 mg L^{-1}, 80 mL) and tetracycline hydrochloride (TC, 30 mg L^{-1}, 80 mL) were studied by using a 300 W Xe lamp as light source (λ > 420 nm, Merry Change, MCPF300B). To ensure the adsorption-desorption equilibrium between the target contaminant and photocatalyst, the suspension was treated by ultrasonic and stirred in the dark for 30 min. During the irradiation process, 3 mL suspension was extracted at given time intervals, then separated by centrifugation at 10000 rpm to

remove the solid photocatalysts. The residual concentration of antibiotic CIP and TC was analyzed by spectrophotometer (UV-vis NIR, TU-1900).

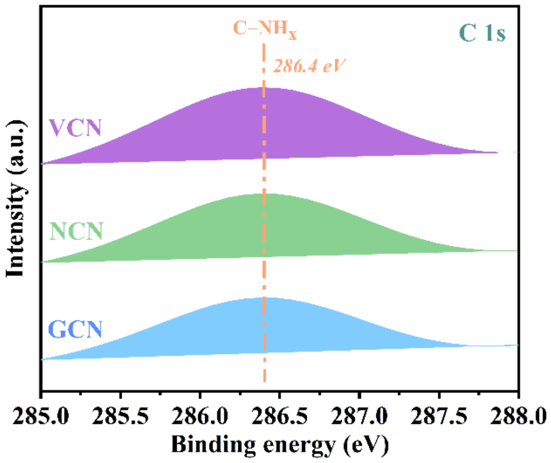

Figure S1. The amplified region for high-resolution C-NHx XPS peak of GCN, NCN and VCN samples.

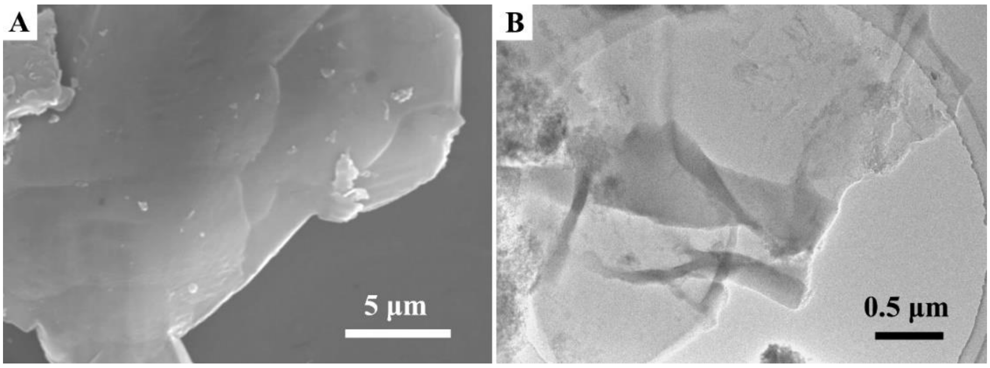

Figure S2. (A) SEM image and (B) TEM image of as-prepared NCN sample.

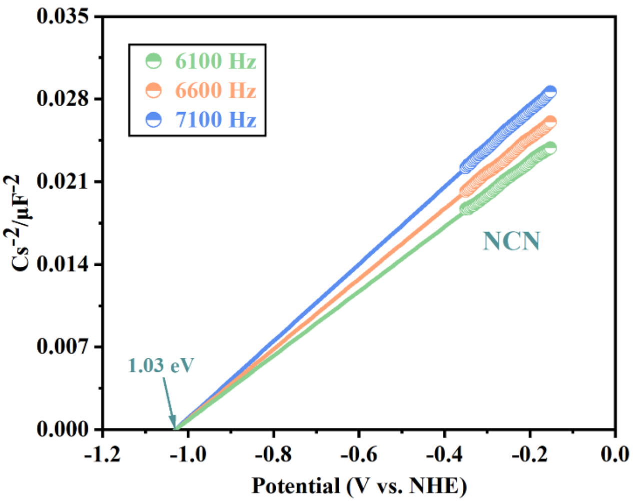

Figure S3. The Mott-Schottky plots with various frequencies of NCN sample.

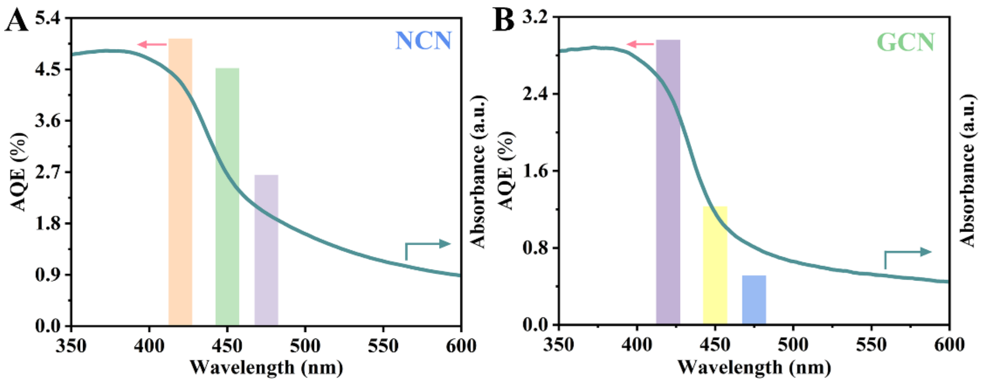

Figure S4. The apparent quantum efficiency of NCN and GCN under the light irradiation wavelengths of 420, 450 and 475 nm.

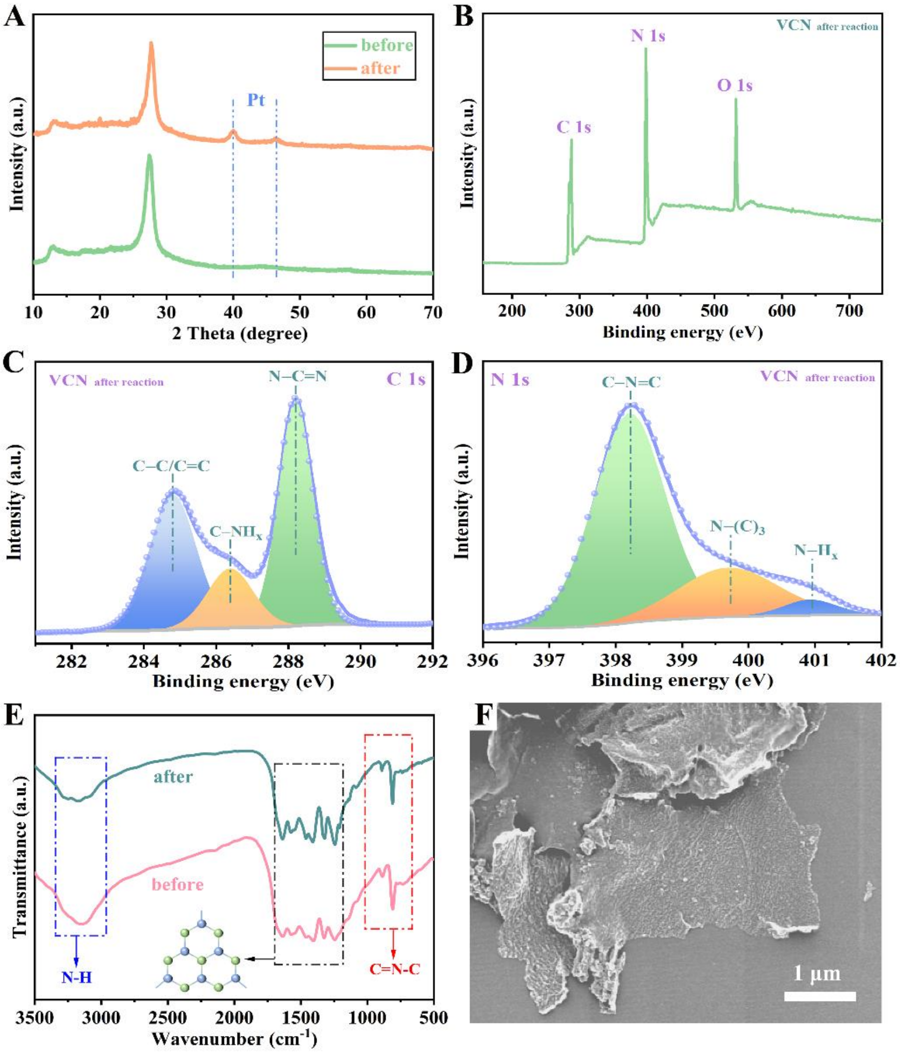

Figure S5. (A) XRD spectra, (B) XPS survey spectra, (C, D) corresponding high-resolution XPS pattern, (E) FT-IR and (F) SEM image of VCN sample after photocatalytic reaction.

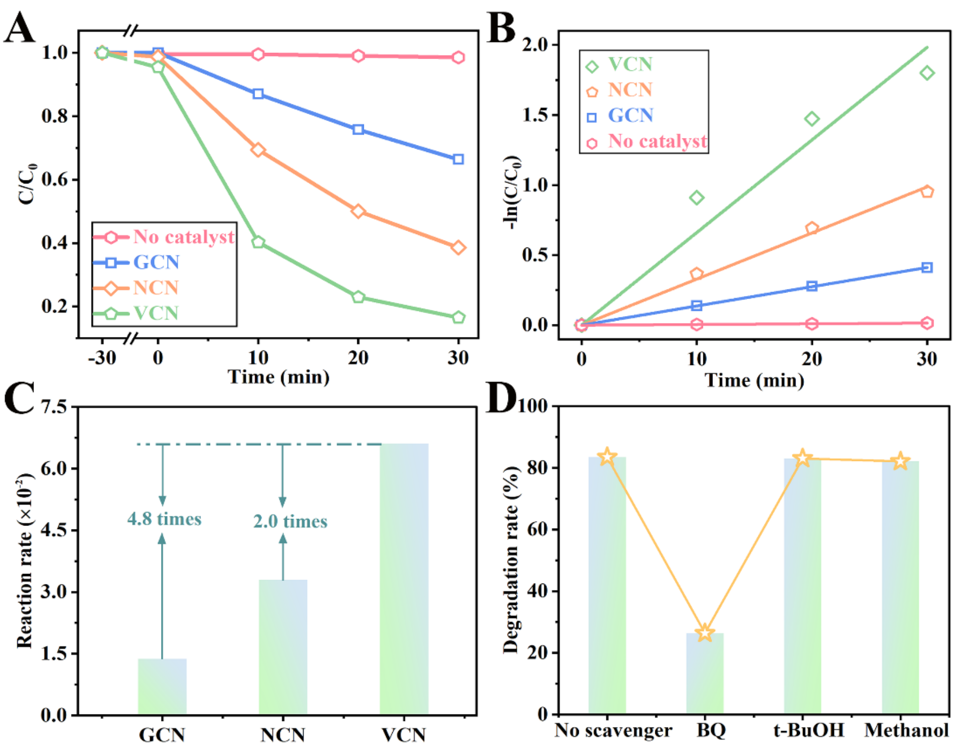

Figure S6. (A) Photocatalytic degradation and (B) corresponding degradation kinetics of TC for GCN, NCN and VCN photocatalysts; (C) Comparison of degradation rate of TC for as-prepared samples; (D) The trapping experiment of TC by using VCN photocatalyst.

Table S1. Pore size, Pore volume and BET surface area of synthesized photocatalysts.

Samples	GCN	NCN	VCN
Pore Size (nm)	—	2.88	2.77
Pore Volume (cm^3 g^{-1})	—	0.0155	0.0414
BET Surface Areas (m^2 g^{-1})	5.5	21.5	59.8

Table S2. ΔG(H*) for the adsorption of H* on g-C_3N_4.

	ε_{ele} (eV)	ZPE+ΔG (eV)	G (eV) 298 K
GCN	-1885.54615888	0	-1885.54615888
H_2	-6.75972273	-0.045115	-6.80483773
H*/GCN	-1885.98201251	0.004576	-1885.97743651

Table S3. ΔG(H*) for the adsorption of H* on structure vacancy defect modified g-C_3N_4.

	ε_{ele} (eV)	ZPE+ΔG (eV)	G (eV) 298 K
VCN	-1789.19311639	0	-1789.19311639
H_2	-6.75972273	-0.045115	-6.80483773
H*/VCN	-1789.69660522	-0.051352	-1789.74795722

Table S4. ΔG(H*) for the adsorption of H* on g-C_3N_4 and supported platinum as a cocatalyst.

	ε_{ele} (eV)	ZPE+ΔG (eV)	G (eV) 298 K
Pt-GCN	-1888.41388585	0	-1888.41388585
H_2	-6.75972273	-0.045115	-6.80483773
H*/Pt-GCN	-1891.60429599	0.131534	-1891.47276199

Table S5. ΔG(H*) for the adsorption of H* on structure vacancy defect modified g-C_3N_4 and

supported platinum as a cocatalyst.

	ε_{ele} **(eV)**	**ZPE+** ΔG **(eV)**	G **(eV) 298 K**
Pt-VCN	-1793.87629646	0	-1793.87629646
H_2	-6.75972273	-0.045115	-6.80483773
H*/Pt-VCN	-1798.33414375	0.215331	-1798.11881275
